# Superamphiphobic coatings based on liquid-core microcapsules with engineered capsule walls and functionality

**DOI:** 10.1038/s41598-018-21957-y

**Published:** 2018-02-26

**Authors:** Malin Nordenström, Anastasia V. Riazanova, Mikael Järn, Thomas Paulraj, Charlotta Turner, Valter Ström, Richard T. Olsson, Anna J. Svagan

**Affiliations:** 1 KTH, Royal Institute of Technology, Department of Fibre and Polymer Technology, Stockholm, SE-100 44 Sweden; 2 WWSC Wallenberg Wood Science Center, Stockholm, SE-100 44 Sweden; 3 RISE Research Institutes of Sweden, Division of Biosciences and Materials, Stockholm, SE-114 28 Sweden; 4 Lund University, Department of Chemistry, Lund, SE-221 00 Sweden; 5 KTH Royal Institute of Technology, Department of Materials Science and Engineering, Stockholm, SE-100 44 Sweden

## Abstract

Microcapsules with specific functional properties, related to the capsule wall and core, are highly desired in a number of applications. In this study, hybrid cellulose microcapsules (1.2 ± 0.4 µm in diameter) were prepared by nanoengineering the outer walls of precursor capsules. Depending on the preparation route, capsules with different surface roughness (raspberry or broccoli-like), and thereby different wetting properties, could be obtained. The tunable surface roughness was achieved as a result of the chemical and structural properties of the outer wall of a precursor capsule, which combined with a new processing route allowed *in-situ* formation of silica nanoparticles (30–40 nm or 70 nm in diameter). By coating glass slides with “broccoli-like” microcapsules (30–40 nm silica nanoparticles), static contact angles above 150° and roll-off angles below 6° were obtained for both water and low surface-tension oil (hexadecane), rendering the substrate superamphiphobic. As a comparison, coatings from raspberry-like capsules were only strongly oleophobic and hydrophobic. The liquid-core of the capsules opens great opportunities to incorporate different functionalities and here hydrophobic superparamagnetic nanoparticles (SPIONs) were encapsulated. As a result, magnetic broccoli-like microcapsules formed an excellent superamphiphobic coating-layer on a curved geometry by simply applying an external magnetic field.

## Introduction

Micro- or nanocapsules that have capsule walls with well-defined morphologies that dictate certain properties and with distinct functionalities in the capsule core, are attractive in a broad scope of applications, including advanced drug delivery, catalysis, imaging, and coatings^1–5^. In nature, a plethora of multifunctional capsule examples are found in the form of plants cells (a natural form of capsule), some of which have adapted their cell-shape and exterior cell-wall morphology to a specific function. For example, within Zinnia leaves, the cells adopt a spongy, star-shape that minimizes cell-to-cell contact, but maximizes gas-exchange via a larger than normal surface area of the cell wall^6^. In the petals of snapdragons, which is a flower, the epidermal cells have a papillae-shape to enrich the color and attract pollinators^6^. The self-cleaning ability of the lotus leaf, the so-called “lotus effect”, is another example of a cell-wall morphology that bears a specific micro to nano-pattern to fit a particular function, that is, enabling a water- and dirt-repellent functionality^7^. Such self-cleaning properties, which are also the focus of the present study, are attractive features to mimic in many daily-life applications. Potential applications are found in self-cleaning windows or moisture protection of corrosive materials, and thus material scientists are presently trying to mimic natural structures or even produce superior structures in order to achieve materials with artificial super-repellent surfaces. Surfaces that repel both water and oils (*γ* < 30 mN m^−1^), that is with static contact angles (SCA) > 150° and roll-off angles < 10°, are termed superamphiphobic surfaces^8^.

So far, the majority of studies on artificial superamphiphobic coatings have focused on exploiting *solid particles* to obtain super-repellent surfaces, but to our knowledge, no studies have focused on liquid-core capsules for the same purpose. There are (only) a couple of studies on water-repellent coatings made from capsules^9–13^. However using liquid-core capsules, more functional features are possible to reach, as substances that depend on liquid mobility can be introduced in the capsule core. For example, capsules could be used to encapsulate and protect oxygen-sensitive molecules such as some fluorescent molecules (liquid mobility = high quantum yield)^14^. Capsules may encompass liquid “healing agents”, which are released upon capsule rupture, triggering autonomic repair^11,13,15^. The liquid core or capsule shell might also contain additional compartments enabling a chain of events or reactions, that occur when triggered using the right stimuli. In a recent study, Keller and Crall demonstrated the potential with liquid-core capsules for “targeted self-healing” using magnetic guidance. The liquid core contained the self-healing components and it was shown that only 1/10 of the healing components was needed due to the magnetic functionality^15^.

In the present study, silica is nucleated and grown *in-situ* as half-spherical nanoparticles on the outer walls of surface-roughened cellulose nanofiber/nanocrystal (CNF/CNC) reinforced precursor capsules. The resulting capsules are hybrid microcapsules with both inorganic (silica) and organic components in the capsule wall. The matrix material in the wall of the precursor microcapsules is a mixture of urethane and urea bonds. The capsule core is filled with oil (hexadecane). By controlling the reaction conditions and additives, we show that silica nanoparticles of different sizes are attained on the outer capsule wall, and that there is a link between the achieved exterior capsule wall morphology (raspberry or broccoli-like) and the SCA of water and oil on a coating-layer made from such capsules. The fabricated “broccoli-like” capsules should not be confused with raspberry-like particles (or capsules) reported in literature, because raspberry-like capsule-coatings demonstrate, at the best, only superhydrophobic properties^16^. To further illustrate the difference, raspberry-like capsules are also fabricated and tested in the present study. The liquid-core of the capsule allows for encapsulation of additional functionalities and in the present contribution this is demonstrated by encapsulating hydrophobic superparamagnetic magnetite nanoparticles, which permits the capsules to adhere to for example curved steel surfaces that can be made magnetic. Such tailored microcapsules, suitable for “targeted superamphiphobicity”, have to our knowledge never been synthesized before. The presented approach opens up for a new type of coatings with water/oil super-repellency, which can be guided to their application without direct physical contact.

## Results and Discussion

### Synthesis and Characterization of Hybrid Microcapsules

Fabrications of superamphiphobic materials with fine structures typically involve complex processing routes such as photolithography^17,18^, candle soot aggregation^19^, and electrospinning^20^. Most of these techniques depend on the specific substrates used during fabrication, and are in some instances destructive to the substrate, thus a more general approach would be useful to coat various substrates^21,22^. One such approach was recently developed by Chen *et al*.^21^. Perfluorinated TiO_2_ microparticles with sword-like nanofeatures were synthesized and superamphiphobicity could be achieved using these solid microspheres, due to the high roughness of the outer particle surface^21^. The microparticles were glued on top of different substrates (glass, cotton, paper etc)^21^. Similarly, Zheng *et al*., prepared superamphiphobic coating on glass, metal, ceramics or wood, that were based on dopamine/silica nanoparticles and where the microscale porous coating structure was prepared via ice templating^22^.

**Figure 1:**
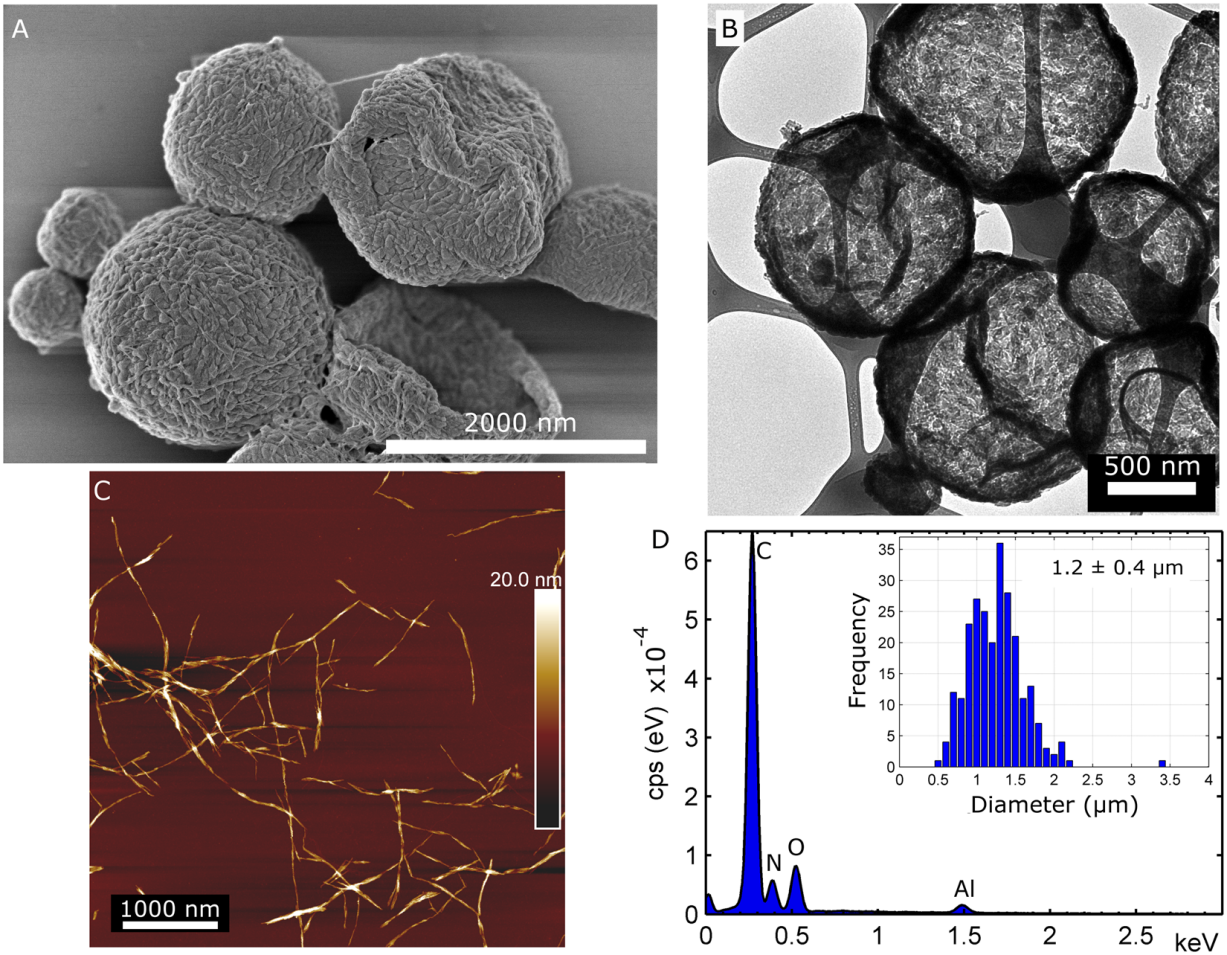
The oil-filled precursor capsules made from toluene diisocyanate, nanocellulose and hexadecane (oil). (**A**) High-resolution SEM image of the precursor capsules. The outer capsule wall has a rough morphology and the presence of nanocellulose is evident. (**B**) TEM micrograph of precursor capsules, demonstrating a hollow core of collapsed capsules. (**C**) AFM image of the nanocellulose used in capsule wall formation. The nanocellulose is a blend of cellulose nanocrystals (CNC) and short cellulose nanofibers (CNF). (**D**) EDS spectrum of precursor capsules. Inset: The size-distribution of the capsules diameters, the average diameter is 1.2 ± 0.4 µm.

A more general approach was also the focus of the present study. Herein, capsules with controlled exterior walls were created by controlling the growth conditions of silica on the outer wall of precursor capsules. The precursor microcapsules, shown in Fig. 1A and B (also Figure S1 in the Supporting Information), had an oil-core cavity (hexadecane) and the microcapsules were in the micrometre size range, see inset histogram in Fig. 1D.

The precursor capsules were fabricated via a direct miniemulsion step by using a combination of short cellulose nanofibers/nanocrystals (CNF/CNC) and toluene diisocyanate. The nanocellulose increases the mechanical properties of the capsule wall and these properties can be fine-tuned^23^. Indeed, cellulose nanofibers (cellulose microfibrils) are the reinforcing components in the walls of plant cells^6^. An AFM image of the used nanocellulose is presented in Fig. 1C. Further contributions from CNF/CNC during capsule synthesis was three-fold. First, the CNF/CNC effectively stabilize (Pickering stabilization) the water/oil interface during the polyaddition reaction at the interface as demonstrated previously. This omits the need for additional surfactants. Secondly, the resulting CNF/CNC based nanocomposite capsules have shown to be quite narrow in size distribution and the processing is simple, thus no time-consuming capsule preparation techniques, such as microfluidics, are necessary. The average diameter for the capsules was 1.2 ± 0.4 µm (inset in Fig. 1D), which is also in accordance with previous results^23^. Thirdly, silica grows as half-spheres on top of cellulosic nanofibers, as shown in a previous study on silica/bacterial cellulose aerogels^24^. This feature facilitates the fabrication of a roughened outer capsule wall. Indeed, to create smooth silica capsules, the underlying surface itself needs to be smooth^25,26^. On the other hand, a rough precursor capsule wall might aid in the development of a roughened outer capsule wall. For this reason, toluene diisocyanate (TDI) was also used to produce a new type of nanocellulose reinforced microcapsule. TDI is a very reactive monomer that readily hydrolyses in water and forms polyurea^27^. FTIR results for the precursor capsule are presented in Figure S2 in the Supporting Information (SI), and showed the presence of both urea^27^ and urethane bonds^28^ in the capsule wall. The urethane bonds are created when the isocyanate reacts with –OH prevalent on the surface of the nanocellulose^23^. The higher reactivity of the monomer produced an even rougher surface of the outer capsule wall, that is, polymer “patches” were created, compare the structure of the present precursor capsules with the nanofibrous structure of capsules made using a less reactive diisocyanate, see previous publication by Svagan *et al*.^23^.

**Figure 2:**
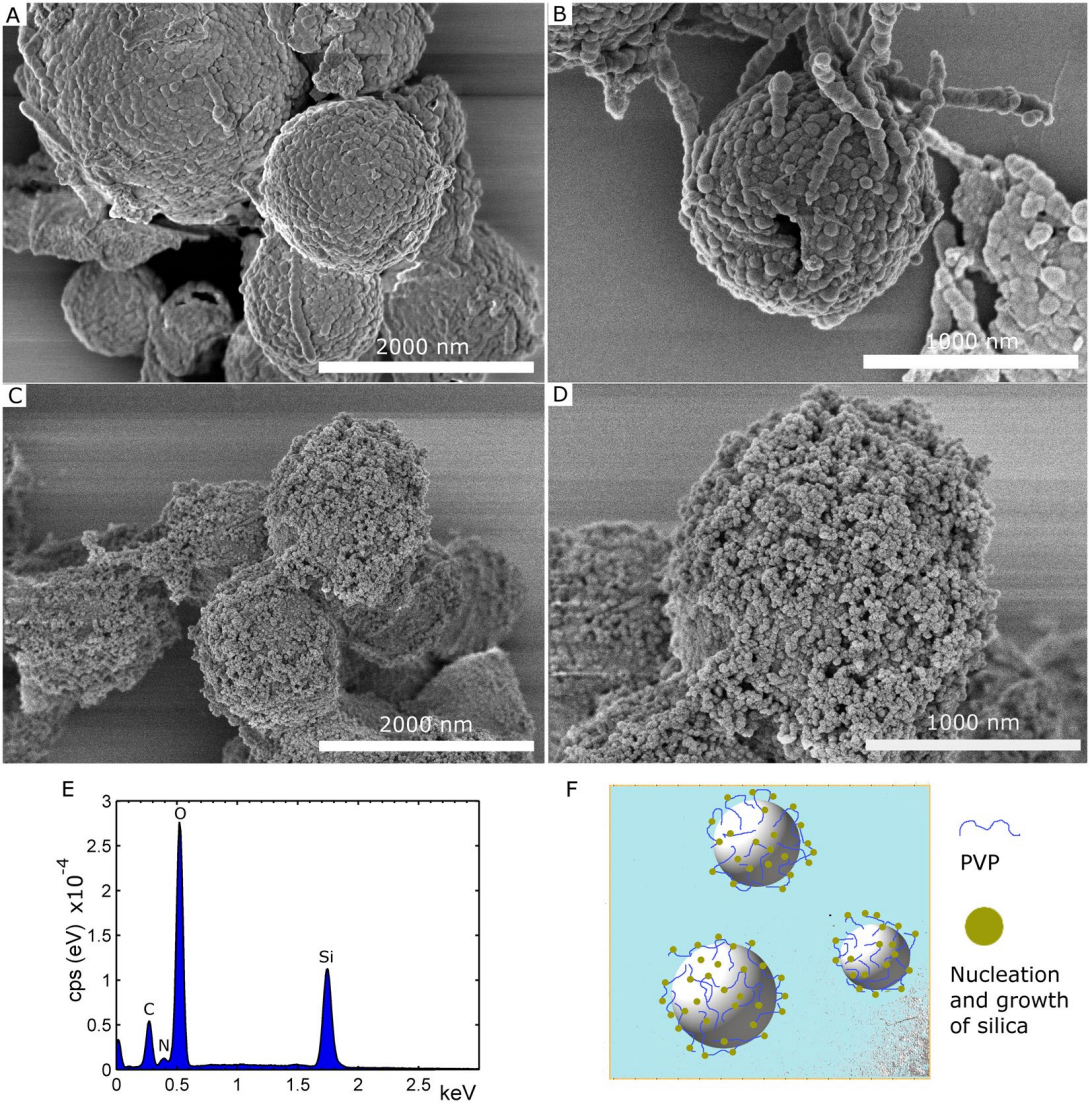
SEM images at two different magnifications of capsules coated with SiO_x_ nanostructures. In (**A**,**B**) the resulting raspberry-like capsules were obtained by adding TEOS to a precursor capsule suspension. In (**C**,**D**) the rougher outer structure of broccoli-like capsules was achieved by adsorbing PVP onto precursor capsules prior to the addition of TEOS. (**E**) EDS spectrum of the raspberry-like capsules shown in (**A**,**B**). (**F**) Proposed model of the adsorption of PVP and subsequent nucleation and growth of silica on the outer wall of broccoli-like capsules shown in (**C**) and (**D**).

To form a nanoparticulate SiO_x_ structure on the outer capsule wall, Tetraethyl orthosilicate (TEOS) and ammonia were added to a suspension of precursor capsules in EtOH/water to start the condensation reactions (sol-gel process), see experimental section. The resulting raspberry-like capsules are shown at two different magnifications in Fig. 2A and B, wherein sphere-like features were formed on the outer capsule wall. These SiO_x_ structures were around 71 ± 15 nm in diameter. Interestingly, most raspberry-like capsules stayed as spheres during the high vacuum conditions during SEM imaging, which demonstrated improved mechanical stability of the capsule wall after SiO_x_ coating formation, compare with the SEM images of the precursor capsules in Fig. 1A and Figure S1 in SI. The energy-dispersive X-ray spectrum (EDS) of the capsules is given in Fig. 2E, confirming the presence of silica. The FTIR spectrum for capsules that were further treated with fluorosilane is found in Figure S3 in the SI. The FTIR spectrum confirmed the presence of both silica and 1H,1H,2H,2H-perfluorooctyl groups.

To further control the structure and achieve finer silica nanoparticles on the outer capsule wall, an additional processing step was needed. This was achieved by pre-treating the capsules with a polyvinylpyrrolidone (PVP) solution in EtOH. After removal of excess PVP polymer, TEOS and ammonia were added and the resulting broccoli-like capsules had a much finer nanoparticle structure on the outer capsule wall, see Fig. 2C and D (also Figure S4 in SI). Even in this case most broccoli-like capsules stayed as spheres during SEM imaging. The obtained silica spheres were much smaller, on average 34 ± 6 nm in diameter, and also more plentiful in comparison to those found on the capsule wall presented in Fig. 2A and B. PVP has previously been used as surface primer coating on top of nanoparticle or template particles (e.g. polystyrene) to promote silica coating and growth by the Stöber process^25,29^, but it has, to our knowledge, never been used in a similar way as it was used herein. In the reported cases, the formed silica grows as a shell of uniform thickness on the template surface. In the present contribution, the layer consisted of silica nanoparticles. Zeta potential measurements showed that the surface of precursor capsules were on average positively charged (ζ = +35 ± 4 mV), which is due to the presence of amine groups (-NH_2_) at the surface that come from hydrolysis of isocyanate groups (-N=C=O) of TDI^28^, see FTIR data in Figure S2. PVP is only slightly negatively charged in EtOH (the present experimental condition)^30^, and this, in combination with the high molecular weight of PVP (Mw = 360 kDa), might contribute to PVP being only loosely associated (via weak electrostatic and hydrogen interactions) with the surface of the precursor capsules. A model picture of the proposed adsorption of PVP on top of the capsules and nucleation/silica growth mechanism is given in Fig. 2F. This would explain why a nanoparticle structure was created instead of the uniform shell coating observed in previous studies (referenced above). Indeed, the added PVP provides more nucleation sites for the silane, which gives smaller and more abundant silica nanoparticles compared to the raspberry-like capsules obtained in the absence of PVP, compare Fig. 2A,B,C,D. SEM observations also indicated that the predominant amount of these silica nanoparticles were homogenously distributed on the surface of the precursor capsules and not observed next to the capsules, suggesting that very few nucleated on PVP that was not coordinated to the precursor surface. Considering that excess PVP was removed by an intermittent washing step (prior to the addition of TEOS and ammonia), this explanation would be in-line with the observations made. The EDS spectrum for the hybrid broccoli-like capsules is found in Figure S5.

### Superamphiphobic performance of a layer of microcapsules with different morphologies

**Figure 3:**
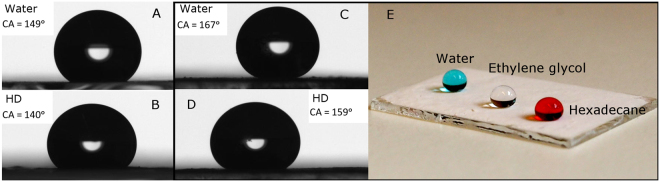
Static contact angle (SCA) of water and hexadecane (HD) on top of two different capsule-coated glass-surfaces, prepared using the capsules in Fig. 2A,B (images 3 A and 3B) and capsules in Fig. 2C,D (images 3 C and 3D). In (**E**) the same coating as in (**C**) and (**D**) is presented with 10 µl drops of MilliQ-water, ethylene glycol and hexadecane.

To evaluate the performance of capsule coatings, a suspension of capsules was first treated with a fluorosilane (to reduce the surface energy) and then deposited on a glass slide to create the coating-layer. When using raspberry-like capsules with outer surfaces as those shown in Fig. 2A and B, the SCA for MilliQ-water was measured to 149° ± 1° and for hexadecane the value obtained was 140° ± 2°, see Fig. 3A and B. These values suggest that these surface-coatings were only strongly oleophobic and hydrophobic.

To attain superamphiphobic properties, the exterior walls of the capsules need to be structured in order to attain a nano-roughened surface that can entrap a sublayer of air. By building a hierarchical capsule surface morphology, and increasing the ratio between micro/nano-surface structure, a larger fraction of air will be present^21^. In this way, there will be a transition from the Wenzel to Cassie state, and a layer of such capsules will display super-repellency of certain liquids^31^. If a coating layer on glass was prepared from broccoli-like capsules as those shown in Fig. 2C and D, that is microcapsules with a hierarchical exterior structure with finer silica nanoparticles, the coating displayed super-repellent properties. The SCA for MilliQ-water was 167° ± 2°, ethylene glycol 163° ± 3° and hexadecane 159° ± 2°, see Fig. 3C–E. The roll-off angle for a low surface tension liquid like hexadecane (γ_HD_ = 27.3 mN m^−1^) was 3° ± 1° (MOVIE_1.avi). When water droplets (γ_water_ = 72.1 mN m^−1^) were deposited on the coating, the droplets instantly rolled off in most cases (roll-off angle < 1°) due to the extreme liquid repellency of the coating. However, due to the somewhat heterogenic nature of the sample some drops did not roll off immediately – but even in these cases the roll-off angles were always less than 6°. A movie is included in the Supporting Information (MOVIE_2.mp4) that shows that water and oil could easily roll down from a tilted coated glass surface without trace of residue, implying low rolling angles and low adhesion. The coatings prepared following the present protocol were satisfactory for demonstrating their superamphiphobic properties, but the robustness of the coatings is rather poor. Therefore, work is ongoing on modifying the protocol for preparation of more durable coating layers.

### Superamphiphobic performance of coatings prepared using magnetic guidance of magnetic broccoli-like capsules

**Figure 4:**
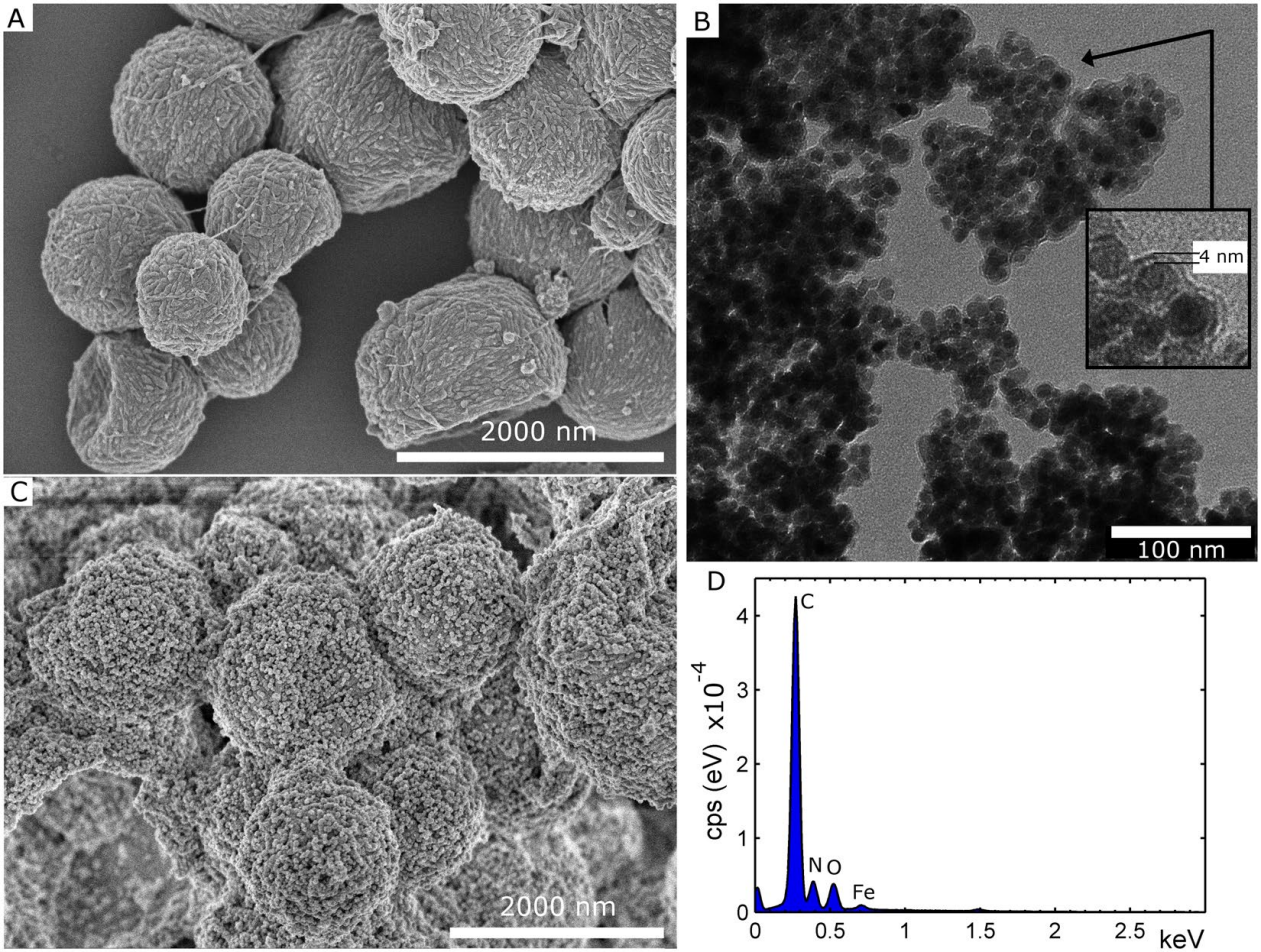
SEM images of magnetic capsules, both precursor capsules (**A**) and after growth of the SiO_x_ nanoparticles (**C**). The capsules contained surface modified SPION. (**B**) TEM image of coated SPION nanoparticles. The nanoparticles were coated with a 4 nm thick OTES coating, rendering their surface hydrophobic. (**D**) The EDS spectrum for the (precursor) capsules in (**A**). The presence of Fe, due to SPION nanoparticles, is evident. The EDS spectrum for capsules in (**C**) is found in Figure S7 in SI.

Fabrication of re-entrant textures on non-flat surface that are uneven, curved or within enclosed geometries is extremely challenging, due to the complexity and limitations of existing top-down and bottom-up approaches^32^. In a recent study by Tricoli *et al*.^32^, superamphiphobic and transparent textures on very complex geometries (tubes) were prepared using a rapid gas-phase concept. In the present contribution, we challenge ourselves further and focus was on entrapping hydrophobic superparamagnetic magnetite nanoparticles (SPIONs) in order to assemble superamphiphobic coating-layers using magnetic guidance. This type of “targeted superamphiphobicity” has, to our knowledge, not been attempted before. The precursor capsules and the resulting broccoli-like microcapsules, which contained hydrophobic SPION nanoparticles, are given in Fig. 4A and D, respectively. The coated SPION nanoparticles are presented in Fig. 4B (coating thickness of 4 nm). TEM images and the X-ray diffractogram of uncoated SPION nanoparticles are presented in Figure S6 in SI. The morphology of both precursor capsules and broccoli-like capsules (silica nanoparticles of 39 ± 8 nm in diameter) did not change in the presence of SPION, which shows that the SPION nanoparticles did not interfere with the capsule formation, compare with images in Figs 1A and 2C.

**Figure 5:**
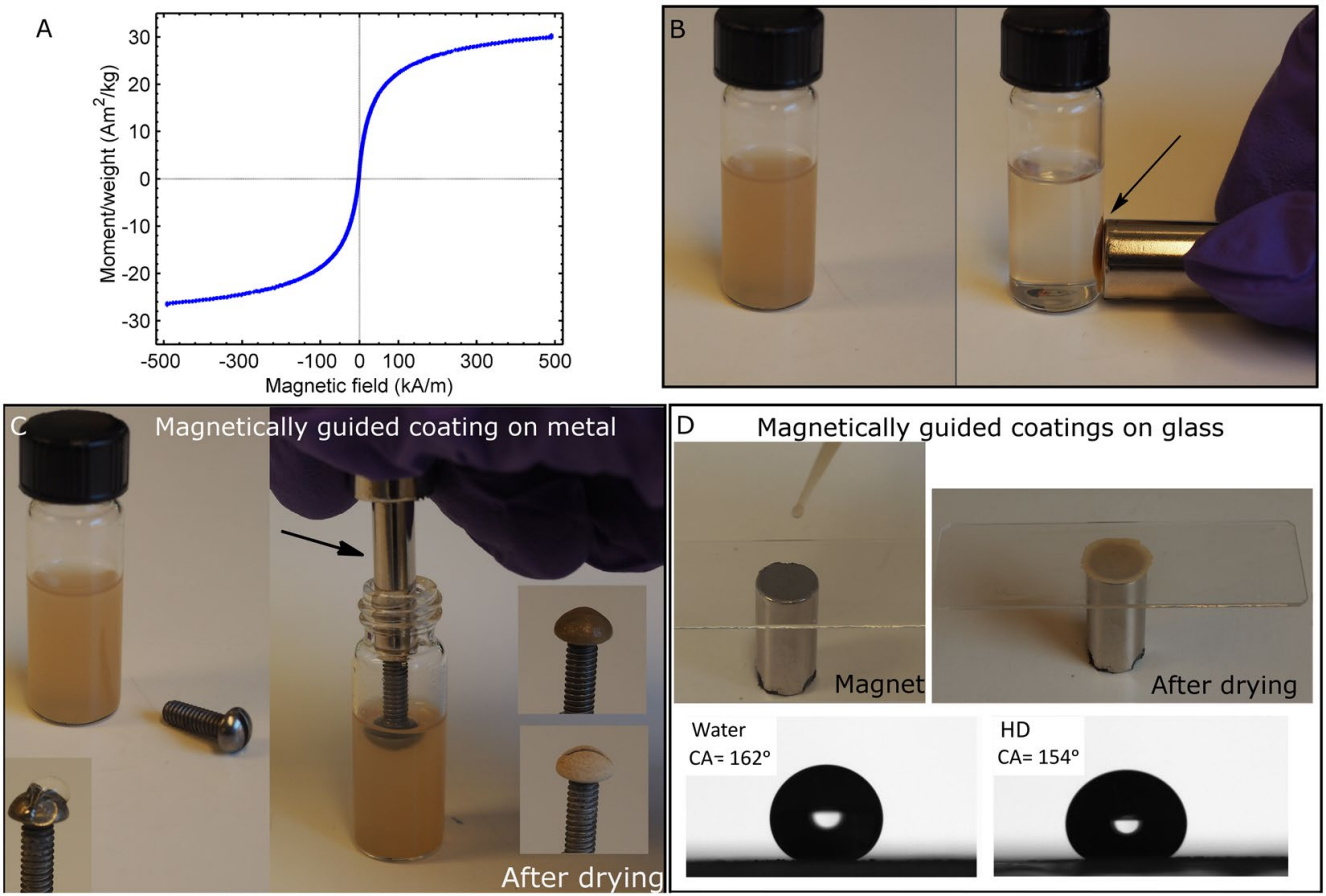
(**A**) Hysteresis loop acquired at room temperature of an aliquot of the broccoli-like capsules charged with the superparamagnetic particles. Magnetization numbers (Am^2^ kg^−1^) have been normalized with respect to nominal weight of magnetite. (B) The magnetic properties of a suspension of broccoli-like capsules in EtOH is demonstrated (the capsule in Fig. 4C, ca. 0.5 wt% capsule suspension), i.e. the capsules accumulate where the magnet is placed (arrow). (**C**) Utilizing the magnetic properties of capsules, it is possible to coat the top of curved steel screw. The screw was attached to a magnet and dipped into the same capsule suspension as in (**B**). The screw is presented prior to coating deposition (left) and with coating in the wet and dry state (right). (**D**) A capsule suspension was added dropwise to a microscopy glass-slide; a magnet was used to accumulate the broccoli-like capsules at a specific site. After assembly, the coatings were treated with a fluorosilane at 50 °C (1 h). The SCA of water and hexadecane was 162° and 154°, respectively.

Additional images of both precursor capsules and broccoli-like capsules with SPION are presented in Figure S8 in the SI. The magnetic properties of broccoli-like capsules are demonstrated in Fig. 5. A magnetic hysteresis loop is shown in Fig. 5A, with a Langevin-like shape and a minute coercivity (the coercivity is well below 100 A m^−1^), which combined substantiates the designation of a superparamagnetic material. The saturation magnetization is however somewhat low (ca. 30 Am^2^ kg^−1^) compared to magnetite. By holding a magnet to the side of a vial containing broccoli-like capsules in EtOH, the capsules will accumulate where the magnet is placed, see Fig. 5B.

Broccoli-like capsules with SPIONs were successfully used to obtain targeted placement of coatings, see Fig. 5C. The steel screw was attached on a magnet (arrow in Fig. 5C) and dipped into the suspension of capsules in EtOH. After some time, a thick enough capsule coating was created and the screw was removed and dried, see Fig. 5C. The challenge with the magnetically guided coating is to obtain a thick enough coating, approx. 70–100 µm. Sometimes, very fine micro cracks could be observed when a coating that was too thick was deposited at once - these aspects will be further addressed in the future. In the supporting information a movie is included demonstrating that both oil and water was able to roll of the coated steel screw (MOVIE_3.mp4).

In addition to the curved steel screw, a circular superamphiphobic spot could also be created on top of a microscope glass slide, by attaching a magnet to the opposite side of the glass during capsule deposition, see Fig. 5D. The SCA was 162° ± 3° for water, 158° ± 2° ethylene glycol and 154° ± 2° hexadecane. Also, droplets of oil and water could easily roll off the circular spot without traces of residue, when deposited on a tilted surface (tilting angle < 10°). However, when these droplets hit the area surrounding the spot, where the glass was exposed, they got stuck and were unable to roll further (MOVIE_4.mp4). This example illustrates that the present concept could potentially be used to create superamphiphobic patterns, utilizing magnetic guidance (targeting) only, that have surface areas around or in-between such patterns that possess different wettability. Such patterned substrates could potentially be beneficial in e.g. fog harvesting devices, microchannel devices, lab-on-chip devices or other droplet manipulation applications^33–35^. Also, the motivation for using microcapsules, instead of solid particles, is that the microcapsules could deliver some additional payload that could be beneficial in e.g. self-healing applications.

## Conclusions

Microcapsules (ca. 1–2 µm in diameter) with tailored exterior surface topologies (roughness), suitable for targeted superamphiphobicity, have been synthesized via a facile co-assembly at the oil/water interface. Using diisocyanate and a blend of nanocellulose only, precursor capsules with a semi-rough exterior wall could be achieved. The outer wall was further used for easy and controllable nucleation and growth of silica nanoparticles (ca. 30–40 nm or ca. 70 nm in diameter), utilizing polyvinylpyrrolidone to assist in the creation of smaller silica particles. The resulting raspberry-like or broccoli-like capsules contained low surface tension oil, hexadecane, in the interior. At the same time, a coating-layer prepared from the broccoli-like capsules repelled water and hexadecane droplets, exhibiting superamphiphobicity. Hydrophobic superparamagnetic nanoparticles were suspended in the liquid capsule-core and the results show that a relatively small amount of solid magnetic material can be used to rapidly localize the functionalized capsules in a concentrated zone, only using a common neodymium magnet. Due to the magnetic property, the capsules could successfully coat a curved surface in a fast and facile way or create a circular pattern on a glass surface, which paves the way for the development of complex (patterned) superamphiphobic coatings. Considering the ease of production and the resulting properties, it is expected that these novel microspheres may also find applications in self-healing materials or future medical and sensory applications. Work is in progress on improving the robustness of the coating-layer.

## Methods

### Materials

Tetraethyl orthosilicate (TEOS), ethylene glycol, octyltriethoxysilane (OTES), polyethylenimine (PEI, Mw = 25 kDa) 1H,1H,2H,2H-perfluorooctyl-trichlorosilane (fluorosilane), hexadecane, ammonium hydroxide (≥25 wt%, aq), polyvinylpyrrolidone (PVP, Mw = 360 kDa), toluene 2,4–diisocyanate (TDI) were purchased from Sigma Aldrich. EtOH (96 vol% and absolute), n-Hexane were purchased from VWR. Olive oil was from Carbonell, Spain (purchased in a domestic grocery shop). The blend of cellulose nanofibers and nanocrystals (CNF/CNC) was the same as in a previous study^23^. Briefly, the nanocellulose was prepared from never-dried bleached sulphite pulp from spruce (14 wt% hemicellulose, <1% lignin, Nordic Paper Seffle AB, Säffle, Sweden) via a modified and mild acid hydrolysis route. The nanocellulose had a surface charge density of 0.031 e nm^−2^. Iron (II) chloride·4H_2_O, iron (III) chloride·6H_2_O were used to prepared the magnetic Fe_3_O_4_ nanoparticles.

### Superparamagnetic magnetite nanoparticles (SPION)

Superparamagnetic nanoparticles (SPION) were prepared according to a previous protocol, using a rapid mixing technique to obtain the nanoparticles^36^. The magnetite nanoparticles were further coated with a hydrophobic OTES coating as described previously^37^. The surface-modified nanoparticles were transferred to hexadecane via two steps; two times washing with EtOH (absolute) and two times with hexane, then the hexane was removed and the nanoparticles were dispersed in hexadecane. Residual solvent (hexane and/or EtOH) was removed by heating the magnetite/hexadecane suspension at 110 °C for 35 min under argon atmosphere and stirring. The final concentration of magnetite in hexadecane was 30 mg mL^−1^.

### Precursor capsule preparation

The precursor capsules were made using a similar protocol as described previously^23^, but using TDI instead of isophorone diisocyanate (IPDI) and no catalyst. TDI was chosen based on its higher reactivity with water compared to IPDI and because the resulting morphology of the capsule wall was rougher. TDI is a toxic monomer and suitable precautions should therefore be taken when working with TDI. Briefly, the 0.5 wt% CNF/CNC suspension was prepared by diluting a stock solution (1.71 wt%) and then ultra-sonicating for 60 s (90%, ½” tip, 750 W Sonics Sonifier Sonics®, USA). This treatment was milder compared to the previous study^23^, and resulted in CNF/CNC that were 7.4 ± 2.3 nm in width (AFM height measurements) and 200–1000 nm in length. An amount of 270 mg TDI in 1.2 g of hexadecane was mixed together with 10 g of the 0.5 wt% CNC/CNF suspension by ultrasonication (80%, ½” tip) for 60 s under ice cooling. In this way oil-in-water emulsions were formed via Pickering stabilization^23^. The polyaddition reaction took place at the droplet’s interface and proceeded overnight under magnetic stirring. The resulting precursor capsule suspensions contained 13 wt% capsules.

Precursor capsules containing hydrophobic SPION nanoparticles were prepared following the steps above but by replacing the pure hexadecane with a SPION/hexadecane suspension (30 mg SPION mL^−1^).

The suspension with precursor capsules with pure hexadecane-cores was used further as it is, however the suspension of precursor capsules with SPION in the oil-core was washed once, by diluting 2.5 mL of the capsule suspension with 22.5 mL MilliQ-water, bringing the capsules to the bottom of the vial by a magnet, removing the supernatant phase and then adding MilliQ-water to obtain the starting volume (2.5 mL).

### Capsules with silica nanoparticle coatings

**Figure 6:**
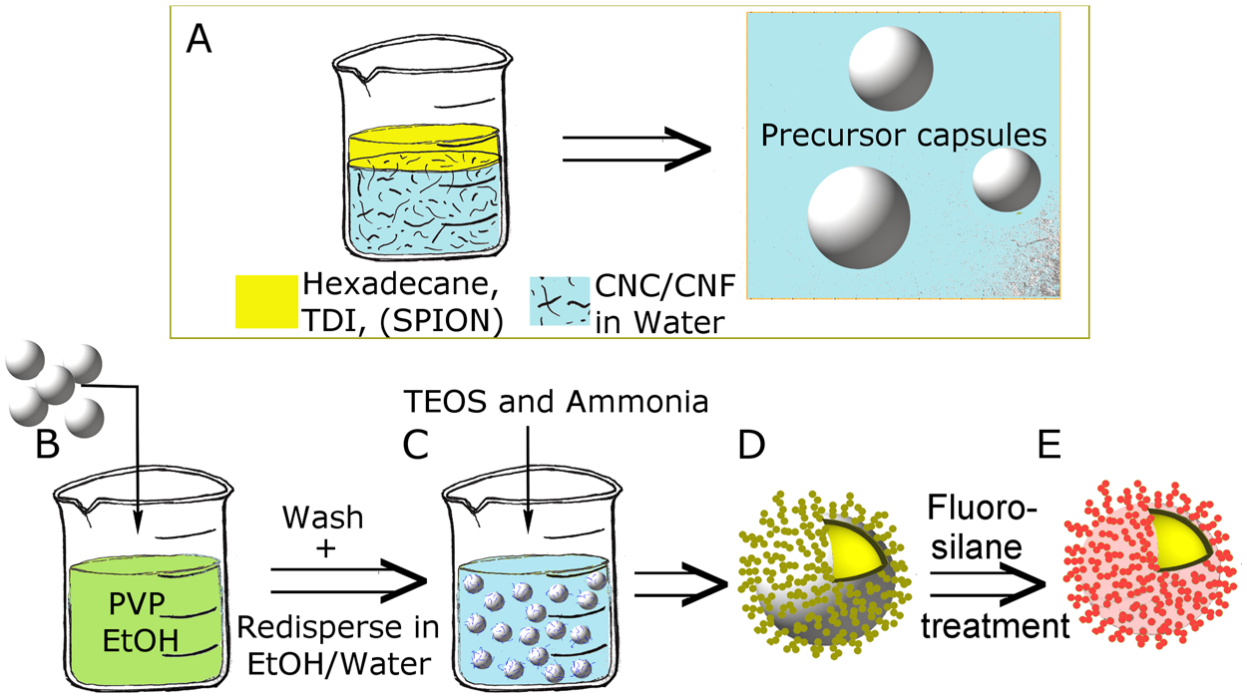
(**A**) Precursor capsules were prepared by ultra-sonicating an oil phase (hexadecane, toluene diisocyanate and with or without superparamagnetic nanoparticles, SPION) with a suspension of CNF/CNC to form an oil in water emulsion. The reaction took place at the oil droplet’s interface. Precursor capsules were added to a PVP solution in EtOH (**B**). The suspension was washed and then dispersed in an EtOH/Water mixture and TEOS and ammonia were added (**C**) to form capsules with silica nanoparticles on the outer capsule walls (**D**). Afterwards, the hybrid capsules were further treated with fluorosilane (**E**).

Broccoli-like capsules were prepared by adding 2 mL of the precursor capsule suspension (with 13 wt% capsules, see details in previous section) to 25 mL of a 7.3 mg PVP per mL EtOH (96 vol%) solution, see Fig. 6. The suspension was left standing for 1 hour under stirring (300 rpm, a shaker was used when the capsules contained SPION nanoparticles. In other cases magnetic stirring was employed). Then the capsules were centrifuged down (4000 rpm, 4 min) and the upper phase was removed and replaced with 2 mL of MilliQ-water and 18 mL of EtOH (absolute). To this 0.6 mL of ammonia and 0.3 mL of TEOS were added and the suspension was stirred overnight (300 rpm, shaker for capsules with SPIONs otherwise magnetic stirring).

Capsules coated with larger silica nanoparticles (Raspberry-like capsules) were prepared in the absence of PVP. Raspberry-like capsules were obtained by adding 2 mL of the precursor capsule suspension (with 13 wt% capsules, see details in previous section) in 18 mL of EtOH (absolute). To this 0.3 mL of TEOS and 0.6 mL of ammonia were added and the reaction proceeded overnight under magnetic stirring (300 rpm).

All resulting capsule suspensions were washed once with EtOH by centrifugation (4000 rpm, 4 min), followed by removal of supernatant and dispersion in EtOH (absolute) to 20 mL. If the capsules contained SPION nanoparticles, the EtOH suspension of capsules was used as is to prepare coatings on glass or metal (see next section below).

For capsules devoid of SPION, the capsules were reacted with the fluorosilane in suspension. The capsules in EtOH were centrifuged down (4000 rpm, 4 min), the upper phase was removed and replaced with 40 mL of n-hexane. This step was repeated twice, but the last time n-hexane was added to give a total volume of 30 mL. The suspension was brought to magnetic stirring (200 rpm) and 100 µL of fluorosilane was added. The reaction proceeded for 15 min and afterwards the modified capsules were washed trice with n-hexane (via centrifugation at 4000 rpm for 4 min, removal of upper liquid phase and dilution to 37 mL with n-hexane). In the last washing step, n-hexane was added to give a total volume of 20 mL.

### Preparation of coatings on glass or metal

Coatings, with capsules treated with the fluorosilane, were prepared by dropping the capsule suspension in n-hexane (ca. 1.8 wt% capsules, prepared as described in previous section) on top of a cleaned (rinsed with EtOH/Acetone) microscope glass-slide (Thermo Scientific), and then dried. Approximately 1 mL of capsule suspension was used to cover an area of 5 cm^2^.

In the case of capsules containing SPION nanoparticles, metal or glass surfaces were coated by magnetic guidance, see Fig. 5C or D. A magnet (standard neodymium magnet with a diameter of 12 mm) was attached to the opposite side of the glass surface or steel screw. The glass was coated by dropping the suspension of microcapsules in EtOH (ca. 1.6 wt% capsules) on the glass surface and then drying. To obtain a coating of good quality, the same glass-slide was coated three times by dropping a 200 µL suspension of capsules each time. The solvent was evaporated before the next layer was applied. The steel screw was coated by dipping it in a diluted capsule suspension in EtOH (dilution to 0.5 wt% capsule suspension) for ca. 3–5 min and then dried, see Fig. 5C. The coated glass and metal screw was placed in a desiccator containing 50 µL fluorosilane in a cup. The desiccator was closed and put in an oven at 50 °C for 1 h for chemical vapor deposition (CVD).

### Characterization

#### Scanning electron microscopy (SEM)

High-resolution images were obtained using a Hitachi SEM S-4800 (Japan) at an accelerating voltage of 1 kV. Samples were deposited on top of Si wafers that were then sputter-coated (Cressington 208HR sputter coater) with a Pt/Pd (60/40) coating prior to imaging. The diameters of the precursor capsules were assessed from 250 capsules (Image J). The diameters of the silica nanoparticles on the outer capsule walls were assessed from at least 60 particles (Image J).

#### Energy-dispersive X-ray spectroscopy (EDS)

Measurements were performed on un-coated capsule samples that were deposited directly on aluminium stubs. Spectra were acquired using an X-Max^N^ 80 Silicon Drift Detector (SDD) from Oxford Instruments (USA) attached to the Hitachi SEM S-4800. The accelerating voltage of 4 kV was used for the EDS analysis.

#### Transmission electron microscopy (TEM)

Images were acquired using a Hitachi TEM HT7700 (Japan) at an accelerating voltage of 100 kV. Samples were deposited onto ultrathin carbon film/holey carbon grids with a 400 mesh copper support.

#### Atomic force microscopy (AFM)

Images were attained with a Multimode 8 AFM (Bruker, USA) in the ScanAsyst mode in air, using a cantilever with a 70 kHz resonance frequency, a 0.4 N m^−1^ spring constant and a 2 nm tip radius (ScanAsyst-Air, Bruker, USA). The samples were prepared by first adsorbing PEI (0.01 wt%, 10 min) onto freshly cleaved mica, rinsing with MilliQ-water, adsorption of the CNC/CNF suspension (0.01 wt%, 10 min), rinsing with MilliQ-water and finally drying.

#### Zeta potential measurements

Measurements were performed with a Zetasizer ZEN3600 (Malvern Instruments Ltd., U.K.) on capsules dispersed in 96 vol% EtOH (0.1 wt% capsule suspension). The zeta potential was calculated by applying the Smoluchowski approximation and the reported value is the average from three measurements.

#### Fourier transform infrared (FTIR)

Spectra were acquired with a Perkin Elmer (Spectrum 100) in the total reflectance mode (attenuated total reflectance accessory). Spectra were collected from 500–4000 cm^−1^ (32 scans, with a resolution of 4 cm^−1^). Prior to measurements, the samples were freeze-dried and then further dried in a vacuum oven at 30 °C for 1 day.

#### Magnetometry

A vibrating sample magnetometer (VSM, Princeton Applied Research 155) was used for magnetic characterization. Hysteresis loops were acquired in ± 500 kA m^−1^. The specimen was an aliquot of capsules containing ca. 1 mg of magnetic nanoparticles.

#### Contact angle and Roll-off measurements

Static contact angles (SCA) were attained with a CAM200 contact angle meter (KSV instruments Ltd, Finland). A 10 μL drop was deposited on the capsule-coated glass-slide using a micropipette and images were recorded and analysed by the software (OneAttension, Biolin Scientific, Sweden). The SCA was measured at five different positions on the surface, and in each position more than 20 images were processed.

The surface tensions of the liquids used in the present study were measured with the same instrument and were 72.1 mN m^−1^ MilliQ-water, 48.4 mN m^−1^ ethylene glycol, 33.4 mN m^−1^ olive oil and 27.3 mN m^−1^ hexadecane.

Methylene Blue and Oil Red O was used to color the water blue and hexadecane red in MOVIE_2, MOVIE_3, MOVIE_4 and Fig. 3E.

Roll-off angles (RAs) were measured with a Dataphysics OCA 40 instrument equipped with a tilting stage. A 10 µL droplet was dispensed on the horizontal surface with a syringe needle after which the sample was tilted at a speed of 0.3° s^−1^. The roll-off angle was recorded as the tilt angle when the droplet was set in motion (see MOVIE_1.avi). The roll-off angle was measured at 8 different positions on the surface.

All measurements were performed at 23 °C and 50% RH.

## Electronic supplementary material

Supplementary Information

Movie_1

Movie_2

Movie_3

Movie_4
