# Supplementary material for: Superamphiphobic coatings based on liquid-core microcapsules with engineered capsule walls and functionality
Source: Sci Rep. 2018 Feb 26;8:3647. doi: 10.1038/s41598-018-21957-y (PMC5832152; doi:10.1038/s41598-018-21957-y)
Supplement: Supplementary file 1 — Supplementary Information [file 41598_2018_21957_MOESM1_ESM.pdf]

Supplementary information

Superamphiphobic coatings based on liquid-core microcapsules with engineered capsule walls and functionality

Malin Nordenström[1,2], Anastasia V. Riazanova[1,2], Mikael Järn[3], Thomas Paulraj[1,2], Charlotta Turner[4], Valter Ström[5], Richard T. Olsson[1], and Anna J. Svagan[1,2,*]

[1]KTH Royal Institute of Technology, Department of Fibre and Polymer Technology, Stockholm, SE-100 44, Sweden
[2]WWSC Wallenberg Wood Science Center, Stockholm, SE-100 44, Sweden
[3]RISE Research Institutes of Sweden, Division of Biosciences and Materials, Stockholm, SE-114 28, Sweden
[4]Lund University, Department of Chemistry, Lund, SE-221 00, Sweden
[5]KTH Royal Institute of Technology, Department of Materials Science and Engineering, Stockholm, SE-100 44, Sweden

Precursor microcapsules

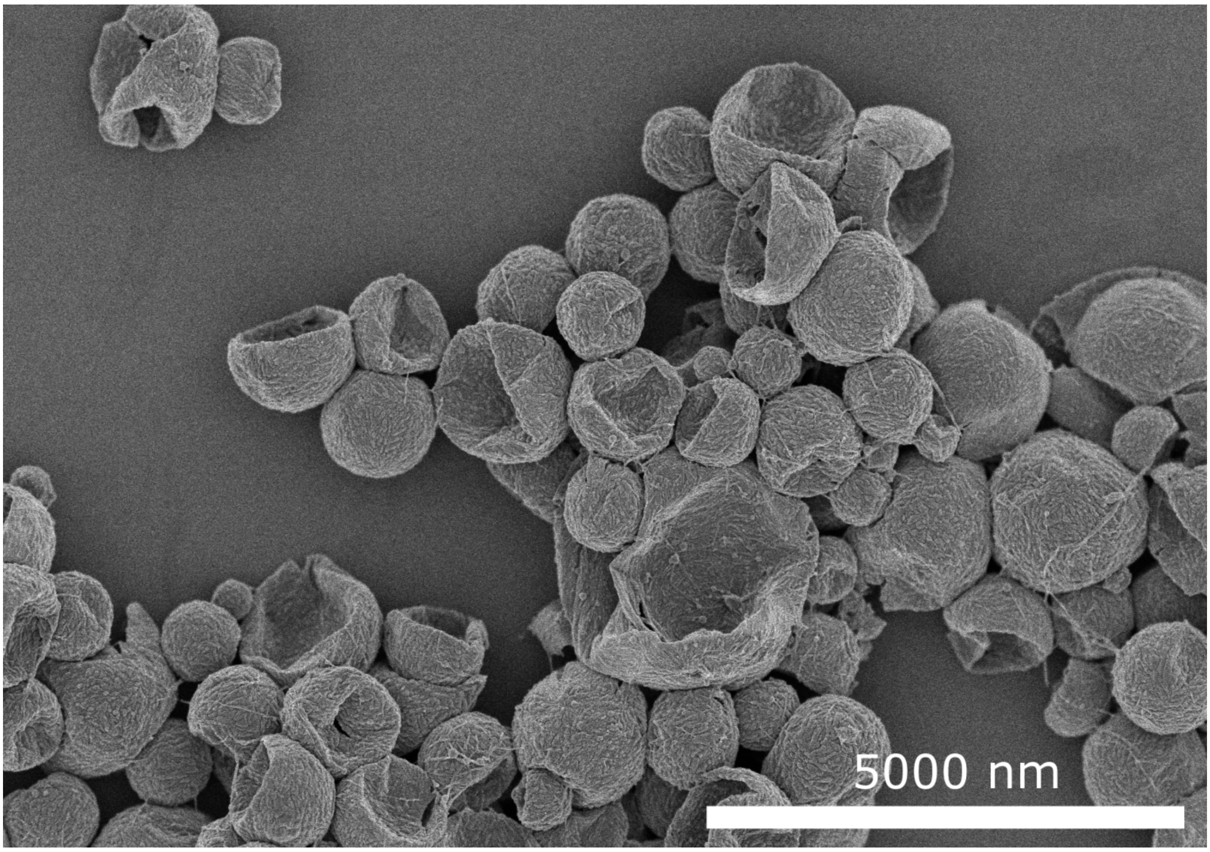

Fig. S1 SEM image of precursor capsules.

An SEM image of the precursor capsules is shown in Fig. S1. Due to the high vacuum conditions in the SEM, the interior of the capsules (hexadecane) evaporated, leaving a deflated and/or partially opened capsules, in accordance with previous results.[1] Previous results also showed that the degree to which the capsules collapse under SEM conditions depends on thickness of the microcapsule wall.[1] The behaviour of the capsules during SEM conditions should however not be confused with their stability at ambient conditions, see previous paper by Svagan et al.[1]

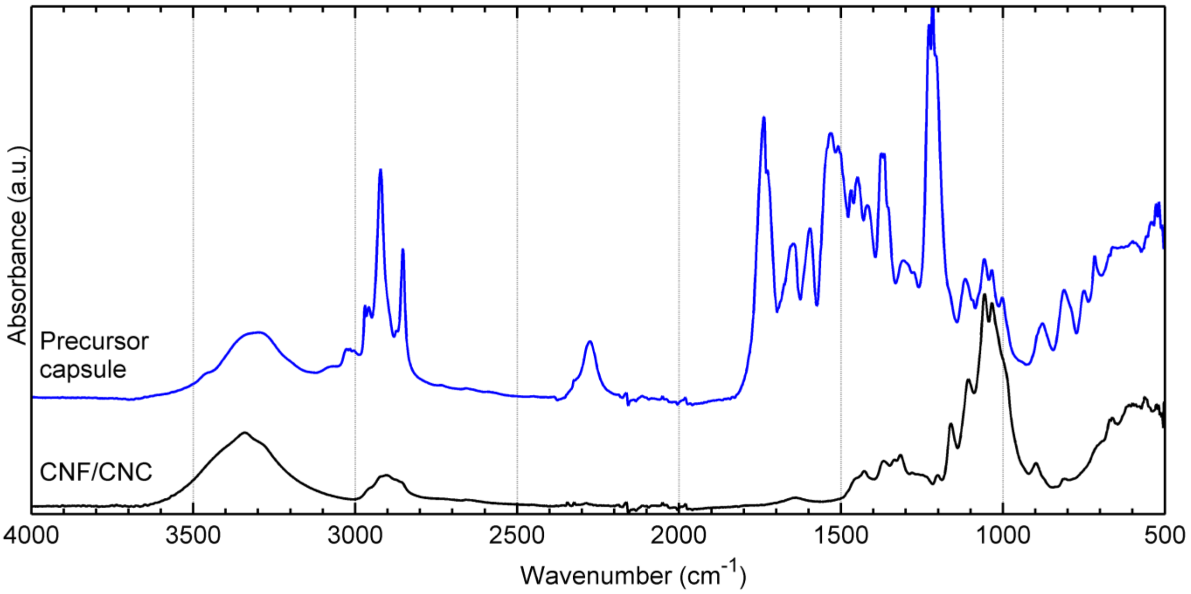

Fig. S2 FTIR data for precursor capsules (blue) and the nanocellulose (black).

The CNF/CNC displayed typical bands for cellulose at 3330 cm^{-1} (-OH stretching), 2900 cm^{-1} (-C-H vibrations in methylene groups) and 1110 cm^{-1} (-C-O in secondary alcohol groups), see Fig. S2.[1] The precursor capsules contained CNF/CNC, urea[2] and urethane bonds[3] in the capsule wall, see Fig. S2. The band found at 1738 cm^{-1} (there is also a shoulder at 1727 cm^{-1}) is due to carbonyl, –C=O, vibrations in urethane bonds formed when the TDI reacts with the –OH groups prevalent on the surface of CNF/CNC.[3] The band at 1645 cm^{-1} is due urea bonds (stretching vibration of carbonyl,-C=O, in urea group) as a consequence of the reaction of TDI with water.[1-3] In the reaction between the isocyanate groups (–N=C=O) of TDI and water, amine groups, $–NH_2$ (and CO_2), are first formed. The amine groups then further react with new isocyanate groups to form the urea bonds. The broad band at around 3300 cm^{-1}, is due to N-H and $-NH_2$ overlapped with the –OH vibration band from cellulose – note there is a shift in this band compared to the band for CNF/CNC (3330 cm^{-1}, -OH stretching). The band at 2273 cm^{-1} indicates that there are still some unreacted isocyanate groups left in (the interior of) the precursor capsule.[1-3]

Capsules with silica on the outer capsule wall

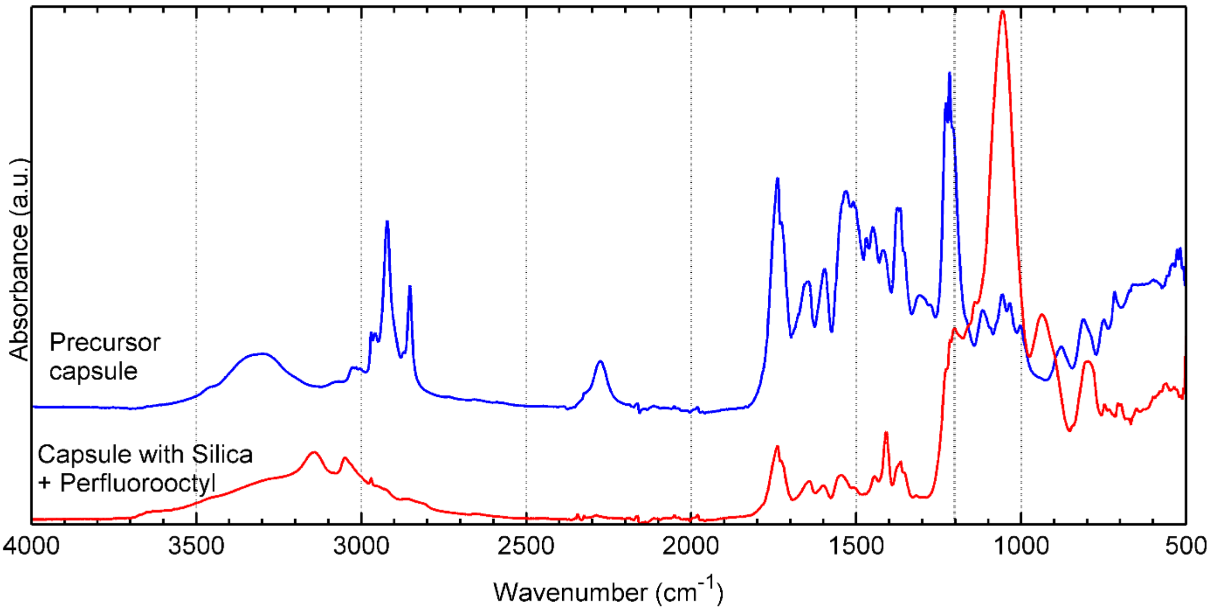

Fig. S3 FTIR data for precursor capsules (blue) and precursor capsules treated with both TEOS and fluorosilane (red). The silica capsule did not contain PVP, that is, the silica is grown directly on the outer precursor wall (raspberry-like capsule type shown in Fig. 2a and 2b in the main manuscript).

After the precursor capsules were treated with TEOS and fluorosilane, new bands appeared at 1056 cm^{-1}, due to asymmetric stretching of linear and branched Si-O-Si,[4] at 937 cm^{-1}, due to Si-OH,[5] see Fig. S3. The band at 1202 cm^{-1} (marked with dotted line in Fig. S3) is due to $–CF_2$ stretching vibration in the perfluorooctyl group.[4] Another interesting observation is that the band for isocyanate groups disappeared, compare with the spectrum for the precursor capsule (Fig. S3). This is perhaps due to the reaction between isocyanate groups and –OH groups attached to Si. The disappearance of reactive isocyanate groups is positive, as unreacted isocyanate groups are toxic.

The FTIR spectrum for broccoli-like capsules that included PVP in the synthesis had bands at the same positions as the spectrum presented in Fig. S3 – the characteristic bands for PVP could not be resolved because they were overlapped by more pronounced bands from other components in the capsule walls.

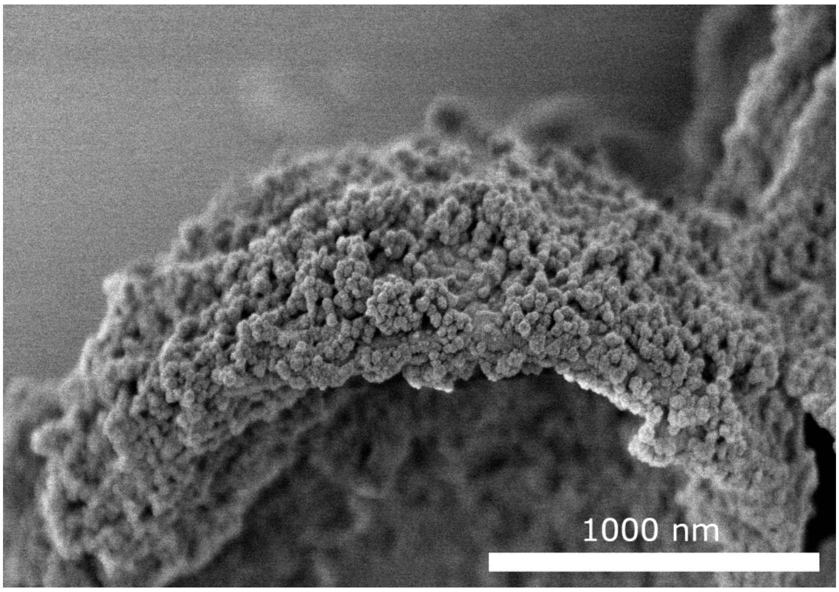

Fig. S4 A close-up of the folded wall of a broken broccoli-like microcapsule. The small silica particles, grown on the outer capsule wall, were obtained using PVP in combination with the rough surface of the precursor capsule.

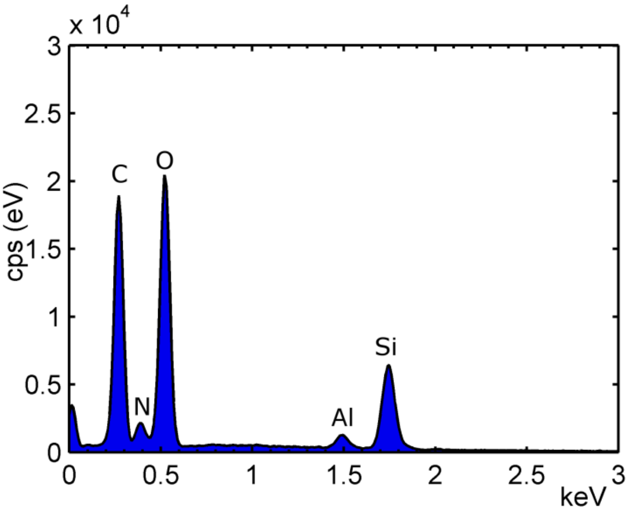

Fig. S5 EDS data for the capsules with smaller silica particles (broccoli-like capsules obtained using PVP to nucleate and grow smaller silica nanoparticles) on the outer capsule wall. (note: Al peak represents a signal from the substrate).

Capsules with magnetic nanoparticles

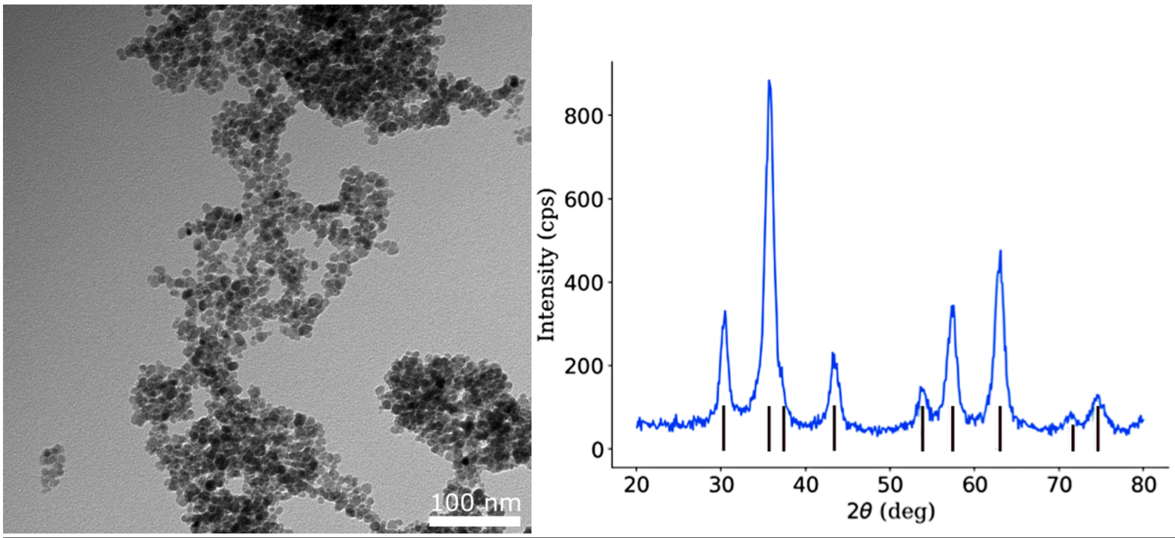

Fig. S6 TEM image of the uncoated SPION nanoparticles and XRD diffractogram of uncoated magnetite nanoparticles.

A TEM image of the uncoated magnetite nanoparticles (ca. 8 nm in size) is presented in Fig. S6. These magnetite nanoparticles had been washed three times with MilliQ-water after synthesis. The nanoparticles were freeze-dried into a powder before X-ray diffraction analysis (XRD, 0.03° step size, Cu K_α radiation (λ = 0.154 nm)).The black bars in the diffractogram in Fig. S6 correspond to the diffraction planes associated with the spinel ferrite structure of magnetite, in accordance with previously reported protocols for the rapid-mixing preparation of magnetite nanoparticles.[6, 7]

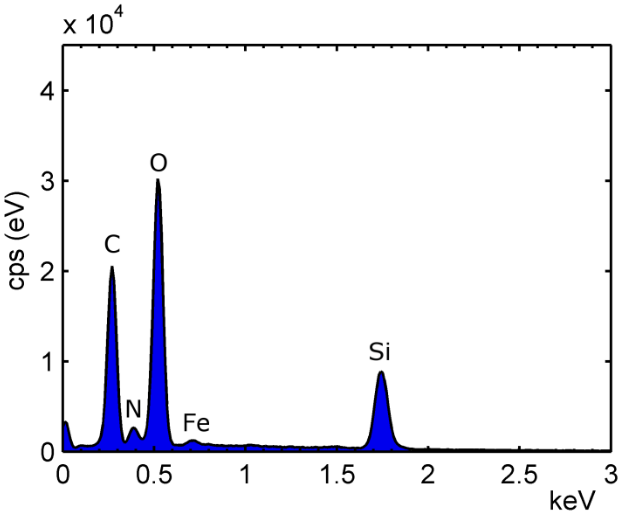

Fig. S7 EDS data for microcapsules containing surface-coated SPION nanoparticles (Fe peak). These capsules contained smaller silica particles (broccoli-like capsules obtained using PVP to nucleate and grow smaller silica nanoparticles) on the outer capsule wall.

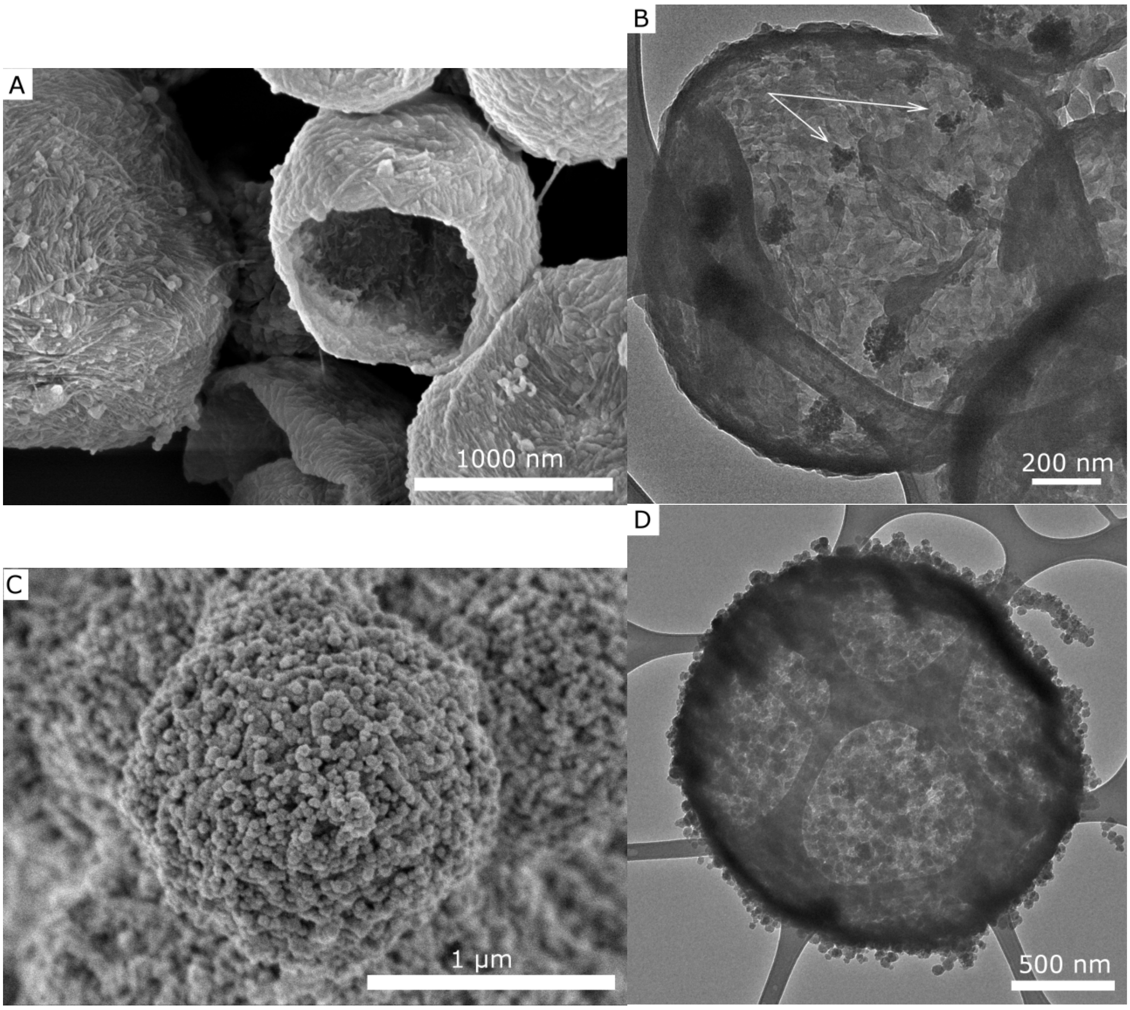

Fig. S8 SEM images (A, C) and TEM images (B, D) of capsules containing SPION nanoparticles. In (A) and (B), the precursor capsules with SPION nanoparticles are shown. In (B) the SPION nanoparticles can be observed in the precursor capsule (arrows). In (C) and (D), broccoli-like capsule are shown. The silica nanoparticles are distributed evenly on the outer surface of the capsules.

Movies included:

MOVIE 1. A hexadecane droplet that rolls off a glass surface coated with a layer of Broccoli-like capsules.

MOVIE 2. The self-cleaning properties of a glass surface coated with Broccoli-like capsules. Exposure to MilliQ-water, ethylene glycol, olive oil and hexadecane.

MOVIE 3. A coated and uncoated metal steel screw exposed to MilliQ-water (blue) and hexadecane (red).

MOVIE 4. The self-cleaning properties of a coated glass surface, where the coating is prepared using magnetic guidance.

REFERENCES

1. Svagan, A.J. et al. Cellulose Nanofiber/Nanocrystal Reinforced Capsules: A Fast and Facile Approach Toward Assembly of Liquid-Core Capsules with High Mechanical Stability. *Biomacromolecules* **15**, 1852-1859 (2014).
2. Li, S.S., Han, H., Zhu, X.L., Jiang, X.B. & Kong, X.Z. Preparation and formation mechanism of porous polyurea by reaction of toluene diisocyanate with water and its application as adsorbent for anionic dye removal. *Chinese Journal of Polymer Science* **33**, 1196-1210 (2015).
3. Baier, G., Musyanovych, A., Dass, M., Theisinger, S. & Landfester, K. Cross-Linked Starch Capsules Containing dsDNA Prepared in Inverse Miniemulsion as "Nanoreactors" for Polymerase Chain Reaction. *Biomacromolecules* **11**, 960-968 (2010).
4. Li, L.X. & Zhang, J.P. Superamphiphobic, Magnetic, and Elastic Silicone Sponges with Excellent Temperature Stability. *Advanced Materials Interfaces* **3** (2016).
5. Kopani, M., Mikula, M., Pinčík, E., Kobayashi, H. & Takahashi, M. FT IR spectroscopy of silicon oxide and HfSiOx layer formation. *Applied Surface Science* **312**, 117-119 (2014).
6. Fang, M., Strom, V., Olsson, R.T., Belova, L. & Rao, K.V. Rapid mixing: A route to synthesize magnetite nanoparticles with high moment. *Applied Physics Letters* **99** (2011).
7. Strom, V., Olsson, R.T. & Rao, K.V. Real-time monitoring of the evolution of magnetism during precipitation of superparamagnetic nanoparticles for bioscience applications. *Journal of Materials Chemistry* **20**, 4168-4175 (2010).
